# Transcriptome deep-sequencing and clustering of expressed isoforms from *Favia* corals

**DOI:** 10.1186/1471-2164-14-546

**Published:** 2013-08-12

**Authors:** Shaadi F Pooyaei Mehr, Rob DeSalle, Hung-Teh Kao, Apurva Narechania, Zhou Han, Dan Tchernov, Vincent Pieribone, David F Gruber

**Affiliations:** 1 The Graduate Center, Molecular, Cellular and Developmental Biology, City University of New York, New York, NY 10065, USA; 2 American Museum of Natural History, Sackler Institute of Comparative Genomics, New York, NY 10024, USA; 3 Department of Psychiatry and Human Behavior, Division of Biology and Medicine, Warren Alpert Medical School, Brown University, Providence RI 02912, USA; 4 John B. Pierce Laboratory, Cellular and Molecular Physiology, Yale University, New Haven, CT 06519, USA; 5 Marine Biology Department, The Leon H. Charney School of Marine Sciences, University of Haifa, Mount Carmel, Haifa 31905, Israel; 6 Department of Natural Sciences, City University of New York, Baruch College, Box A-0506, 17 Lexington Avenue, New York, NY 10010, USA

**Keywords:** K-mer, Contig, Open reading frame, Fluorescent protein, Blast, Clustering, High-throughput sequencing, Illumina paired-end, Coral

## Abstract

**Background:**

Genomic and transcriptomic sequence data are essential tools for tackling ecological problems. Using an approach that combines next-generation sequencing, *de novo* transcriptome assembly, gene annotation and synthetic gene construction, we identify and cluster the protein families from *Favia* corals from the northern Red Sea.

**Results:**

We obtained 80 million 75 bp paired-end cDNA reads from two *Favia* adult samples collected at 65 m (*Fav1*, *Fav2*) on the Illumina GA platform, and generated two *de novo* assemblies using ABySS and CAP3. After removing redundancy and filtering out low quality reads, our transcriptome datasets contained 58,268 (*Fav1*) and 62,469 (*Fav2*) contigs longer than 100 bp, with N50 values of 1,665 bp and 1,439 bp, respectively. Using the proteome of the sea anemone *Nematostella vectensis* as a reference, we were able to annotate almost 20% of each dataset using reciprocal homology searches. Homologous clustering of these annotated transcripts allowed us to divide them into 7,186 (*Fav1*) and 6,862 (*Fav2*) homologous transcript clusters (E-value ≤ 2e^-30^). Functional annotation categories were assigned to homologous clusters using the functional annotation of *Nematostella vectensis*. General annotation of the assembled transcripts was improved 1-3% using the *Acropora digitifera* proteome. In addition, we screened these transcript isoform clusters for fluorescent proteins (FPs) homologs and identified seven potential FP homologs in *Fav1*, and four in *Fav2*. These transcripts were validated as bona fide FP transcripts via robust fluorescence heterologous expression. Annotation of the assembled contigs revealed that 1.34% and 1.61% (in *Fav1* and *Fav2*, respectively) of the total assembled contigs likely originated from the corals’ algal symbiont, *Symbiodinium spp*.

**Conclusions:**

Here we present a study to identify the homologous transcript isoform clusters from the transcriptome of *Favia* corals using a far-related reference proteome. Furthermore, the symbiont-derived transcripts were isolated from the datasets and their contribution quantified. This is the first annotated transcriptome of the genus *Favia*, a major increase in genomics resources available in this important family of corals.

## Background

With the advent of Next-Generation Sequencing (NGS) technology, genomic data acquisition has become much easier, especially for non-model organisms [1]. The generation of transcriptomes from non-model organisms has also benefitted from NGS advances. Transcriptomic datasets can facilitate genome annotation, single-nucleotide polymorphism (SNP) analysis [2], marker development for population genetic and adaptive evolutionary studies [3], as well as functional classification [4] in non-model species. The application of transcriptome deep sequencing in metabolic pathway reconstruction and gene marker development has already shown great promise in *Camellia sinesis*[5], *Cicer arietinum*[6], *Sphenodon punctatus*[7], and *Anopheles funestus*[8].

This method is also valuable for relatively understudied species, such as *Favia* corals. Though corals are high in economic and ecological value, limited genomic resources are available, largely because samples are difficult to obtain. Because NGS requires only small amounts of animal tissue, it is possible get large amounts of information from very small samples (1–2 coral polyps). Recently, anthropogenic threats such as climate change, metal pollution and oceanic acidification [9] have led to rapid declines in worldwide coral populations, lending increased urgency to the need for genomic data. Detailed understanding at the genomic and transcriptomic level will allow for the development experimental studies to assess how the intensity and frequency of disturbances affects coral health and abundance.

Several studies have reported NGS long reads transcriptome sequencing of coral species such as *Acropora millepora*[10,11] and *Pocillopora damicornis*[12]. In addition, other recent studies have used the Short Sequence Reads (SSR) platform [13], or combined SSR and long reads approach to explore whole transcriptome modulation in response to low pH in adult *Pocillopora damicornis*[13]*,* and in early life stages of *Acropora millepora*[14]. Yet, these coral clades are quite phylogenetically divergent from *Favia*[15].

This *Favia* genus is one of the most widely and uniformly distributed of all coral genera and is phenotypically presented as massive, dome-shaped and flat. In many cases *Favia* species exhibit cryptic species complexes and their phylogeny has been parodied as being a “Bigmessidae” [16]. *Favia* are in the *Faviidae* family that contains twenty-four genera, more than any other coral family [17]. *Faviidae* is one of the highly fragmented families and Indo-Pacific members appear to be distinct from Atlantic counterparts. Therefore, adding more molecular markers to resolve their phylogeny will add further resolution to coral systematics.

**Figure 1:**
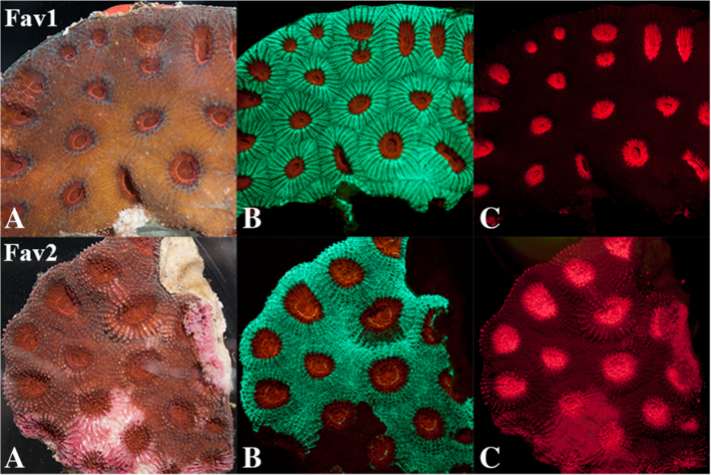
**White light and fluorescent macrophotography of scleractinian coral samples.** Samples of *Favia sp.* were placed in a narrow photography tank against a thin plate glass front. Fluorescent macro images (13.1 megapixel; Nikon D300S) were produced in a dark room by covering the flash (Vivitar 185) with interference bandpass excitation filters (Semrock, Rochester, NY). Longpass and bandpass emission filters (Semrock) were attached to the front of the camera. **A)** White light image; **B)** ex. 450–500 nm; em. 514LP; **C)** ex. 500–550 nm, em. 555 LP.

We sequenced and assembled 58 Mbp of Illumina cDNA reads from two coral *Favia* samples, termed “*Fav1*” and “*Fav2,*” that were collected at 65 m in the northern Red Sea (Figure 1). Reads were assembled into contigs and annotated to: 1) identify protein family clusters using the proteome of *Nematostella vectensis* as a reference; 2) assign functions to the protein family clusters using *Nematostella vectensis* GO, InterPro and KOG functional annotation*;* 3) identify homologous proteins in *Acropora digitifera* using sequence-based similarity searches; 4) identify symbiont-derived contigs in our assembly; and 5) conduct phylogenetic assessment using three molecular markers (Cytb, COI, 28S) and eleven full-length fluorescent proteins. The resulting data provide a valuable resource for future studies in *Faviids* and other corals.

## Results and discussion

### *De novo* assembly

Holobiont cDNA libraries were synthesized from the RNA of two individual adult *Favia sp.* collected from the Gulf of Eilat in the Red Sea. Illumina runs performed on each separate, normalized, cDNA pool generated approximately 80 million reads per sample with average quality scores > Q20 at each base. The first step of assembly was carried out with ABySS [18,19], a *de Brujin* graph assembler. In order to recover transcripts across a range of expression levels, we carried out assembly across a range of k-mer values. Transcripts with low depth (i.e. weakly expressed) are best recovered with low k-mer values, while high depth (i.e. highly expressed) transcripts are best recovered with high k-mer values [20]. Using a range of k-mer values also allows for the identification of expressed splice variants arising from a single gene. As the Illumina read length was set to 75 bp, we chose initial k-mer values ranging from 29 to 45 bp for each sample run.

**Figure 2:**
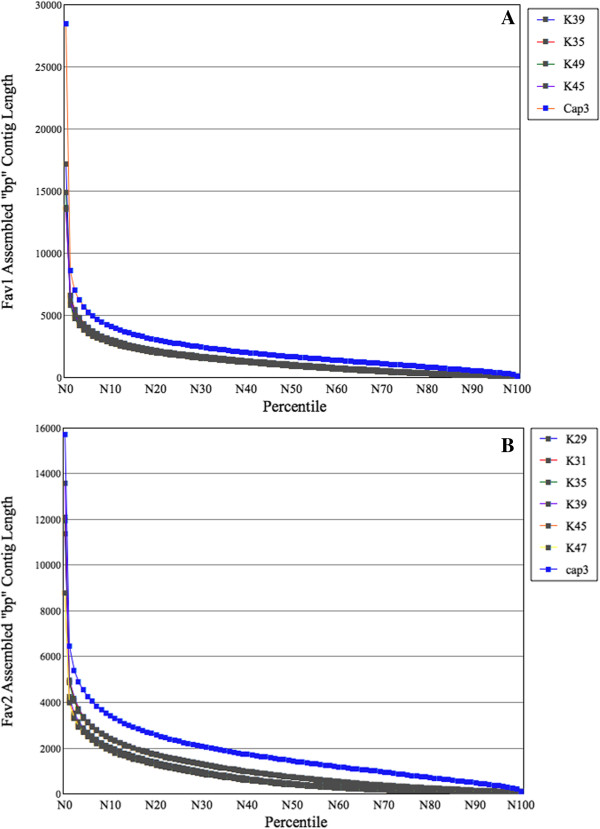
**Contig length improvement after using CAP3.** N50 (50% of the length of the assembled sequences) is a parameter to assess the contig length distribution **(A)***Fav1* contig length and N-values relationship. The thin lines represent the values for k-mer 35, 39, 45. The N50 length values were 1027, 1009, 949 bp, respectively. The line with cross represents the N-values after using CAP3, with N50 length of 1665. **(B)***Fav2* contig length and N-values relationship. The N50 length values for k-mer 39, 45, 49 were 453, 408, 391 bp, respectively. The N50 length values for k-mer 29, 31, 35 were 742, 734, 721 bp, respectively. The line with cross represents the N-values after using CAP3, with the N50 length of 1439 bp.

We evaluated various assembly parameters (e.g., total number of contigs, contigs longer than 100 bp, N50 length, and average contig length) as a function of k-mer length. The three k-mer values (35, 39, 45 for *Fav1* and 31, 35, 39 for *Fav2*) with the highest N50 length [21] were selected as being most informative. In each sample, we eliminated contigs shorter than 150 bp [20] in the two k-mer assemblies with the shortest median contig length, but kept all the contigs in the assembly with the longest median contig length in order to retain any information useful for bridging in the subsequent assembly steps. Within each sample, the three k-mer assemblies were then combined, and the combined contigs were assembled with CAP3 (using default parameters), which computes overlaps to correct errors in constructing contigs and generates consensus sequences for contigs [22], thus eliminating redundant contigs. It has been suggested that assembly of ABySS followed CAP3 yield better contigs [19]. As a result, the N50 length distribution improved after using CAP3, and the best N50 values increased from 1027 to 1665 in *Fav1*, and 742 to 1439 in *Fav2* (Figure 2). The final assembled datasets, which were used for all subsequent analyses, contained 58,848 sequences in *Fav1* and 62,469 sequences in *Fav2*. The N50 values of these two datasets were higher than previous short-read publications [5,7,23] (675 bp, 1438 bp, 506 bp, respectively), suggesting that the quality of our data was comparable to results in other non-model species (For all commands and parameters, see Additional file 1: File S1).

### Homologous clustering of expressed coral transcripts

After using the EMBOSS package [24] to generate all possible open reading frames (ORFs) from stop to stop for each assembled contig, the resulting predicted ORFs were searched for sequence similarity against the *N. vectensis* proteome [25], using reciprocal BlastP (E-value ≤2e^-30^) [26] (Script 1). For the 519,766 predicted ORFs longer than 150 bp, 12,141 unique ORFs in *Fav1* showed considerable sequence similarity to 7,186 existing protein sequences in *N. vectensis*. Similarly, 12,425 unique ORFs in *Fav2* showed similarity to 6,862 *N. vectensis* protein sequences (Additional file 2: File S2, Additional file 3: File S3). The top Blast hits for each sample were saved in a pre-clustering list using a Perl script (Script 2; Output files reported in Additional file 4: File S4, Additional file 5: File S5). These lists were then used in TRIBE-MCL [27] to identify homologous protein family clusters in a comprehensive and uniform way (Additional file 6: File S6, Additional file 7: File S7). The main clustering parameter, inflation value (r), was selected as default (r = 2.5). *Fav1* and *Fav2* had similar numbers (7,186 and 6,862, respectively) of protein family clusters homologous to unique *N. vectensis* proteins. These clusters were subjected to further functional annotation.

In order to evaluate the completeness of our annotation using *N. vectensis* as the reference as opposed to using another available Cnidarian non-annotated proteome (*A. digitifera)*, we applied a newly-developed completeness metric [28] (*In prep.*) to determine the proportion of the reference proteome covered by our sets of assembled transcripts. Only those ORFs with length coverage ≥80% of the matched protein from the *N. vectensis or A. digitifera* proteome were included. Completeness measurements in *Fav1* and *Fav2* compared to *N. vectensis* were 29.54% and 28.20%, respectively; when the same procedure was carried out using the unannotated proteome of *A. digitifera* as a reference (23,677 ORFs downloaded from http://marinegenomics.oist.jp/genomes/downloads?project_id=3. This showed an improvement of only 1-3%, thus validating our usage of *N. vectensis* as a reference proteome (Additional file 8: Table S1).

### Functional annotation and characterization of the isoform clusters in *Fav1*

To identify the putative function of 7,187 isoform clusters, Gene Ontology (GO) and protein domain (KOG, InterPro) searches were performed using the functional annotation of the *N. vectensis*. (Data downloaded from the JGI genome project http://genome.jgi-psf.org/Nemve1/Nemve1.download.html). The clusters were assigned gene names based on the gene name annotation of the best Blast match for the sequences (Additional file 9: File S8). This process successfully assigned gene names for 6,632 (92.27%) clusters using GO term, KOG description, and InterPro description. Among 12,141 annotated best hits, 11,411 (93.98%) gene names were assigned to sequences*.* These provide a rough estimate of the number of different genes expressed in *Fav1* libraries. Broadly, the putative homologs of genes involved in various cellular processes and pathways found to be functionally conserved.

Based on GO terms assignment to clusters, a total of 4,678 (65%) clusters were assigned at least one GO term, among which 11% were assigned at least one GO term in biological processes, 48% in molecular function and 6% in cellular component category (Additional file 10: Figure S1). Among the various biological processes, protein metabolism, and electron transport were mostly highly represented (Table 1). Protein metabolism is also highly represented in other transcriptome characterization studies [6,7,29].

**Table 1:** **Top 30 frequent annotated functions of homologous protein clusters in *Fav1***

**Top frequent GO-annotated homologous protein clusters in *Fav1***		
**Go categories/Description**	**Count**	**Percentage**
Total clusters	7,187	
Total (GO-annotated)	4,677	65.1%
**Molecular function**	3,477	48.37%
1-Nucleic acid binding	241	5.15%
2-Protein kinase activity	218	4.66%
3-DNA binding	208	4.45%
4-Catalytic activity	173	3.70%
5-Calcium ion binding	158	3.38%
6-ATP binding	129	2.76%
7-Protein binding	119	2.54%
8-GTP binding	114	2.44%
9-Transporter activity	96	2.05%
10-Structural constituent of ribosome	82	1.75%
**Biological process**	776	16.59%
1-Metabolism	122	2.61%
2-Electron transport	88	1.88%
3-Intracellular signaling cascade	54	1.15%
4-Proteolysis and peptidolysis	48	1.03%
5-Protein folding	47	1.00%
6-Protein modification	31	0.66%
7-Cell adhesion	29	0.62%
8-Intracellular protein transport	26	0.56%
9-Carbohydrate metabolism	21	0.45%
10-Regulation of cell cycle	18	0.38%
**Cellular component**	424	6%
1-Ubiquitin ligase complex	68	1.45%
2-Integral to membrane	58	1.24%
3-Membrane	58	1.24%
4-Nucleus	46	0.98%
5-Intracellular	41	0.88%
6-Cytoplasm	26	0.56%
7-Cytoskeleton	24	0.51%
8-Nucleosome	16	0.34%
9-Chromatin	10	0.21%
10-Extracellular region	8	0.17%

Top 30 high frequent annotated homologous protein clusters under cellular component, molecular function and biological processes. Full annotation included in Additional file 9: File S8.

According to assigned KOG descriptions to *Fav1* clusters, a total of 6,326 (88%) clusters were assigned at least one KOG description. However, this was 4,489 (62.45%) with InerPro description assignment. This implies that the KOG description was most useful in assigning domain description to our dataset compared to InterPro. The top most frequently detected domain, associated with KOG and InterPro assignment, include conserved domain associated with predicted E3 ubiquitin ligase, fibrillins and related proteins containing Ca2 + −binding EGF-like domains, FOG: Zn-finger, GPCR Rhodopsin, and Ras GTPase superfamily. One of the utilities of domain annotation is that it provides quick access to homologs of genes with known roles in intercellular signaling pathway. The representation of genes involved in intracellular signaling pathway was very similar to that of *A. millepora*[10]. However, a few families showed the events of expansion (for example, Patched, Hepatocyte nuclear factor 4 and Activin-like kinase) and contraction (for example, Notch-delta, Frizzled, Wnt etc.) indicating their functional significance (Table 2).

**Table 2:** **Intracellular signaling pathway genes annotated in *Fav1***

**Intracellular signaling pathway proteins annotated in *Fav1***		
**Pathway**	**Protein name**	**Sequences (n)**
Hedgehog	Patched	27
	Sonic	2
	Fused	1
	Receptor activity (IFRD-C)	1
	DUF699	2
	Smoothened	12
JAK/STAT	STAT protein	1
NFKB/Toll	Nuclear factor NF-kappa-B	1
	Intermediate in Toll-signaling	1
	Toll-like receptor	1
NHR	Hepatocyte nuclear factor 4	2
Notch	Notch	4
	TACE	3
RTK	RTK signaling protein	1
TGF-beta	Activin-like kinase	8
	SMAD	9
	TGF-beta-receptor	1
WNT	Frizzled	9
	Wnt	2

Further, we identified major transcription factors encoding transcripts. In comparison to *A. millerpora*[10], the represented genes were somewhat similar. However, a few families were newly reported in our dataset (For example, HMG box, T-box, ETSDomain, MADS) (Table 3).

**Table 3:** Major transcription factor families identified by conserved domain annotation

**Transcription factors identified by KOG/InterPro/GO annotation in *Fav1***	
**Sequence description**	**Sequences(n)**
CBF	1
Transcriptional Coactivator P50	1
Transcriptional Coactivator P100	6
Transcriptional Coactivator CAPER	2
Homeobox domain	7
HSF-type DNA-binding	1
P53 DNA-binding domain	2
NF-X1-type zinc finger protein	3
Dimerization partner (TDP)	2
Fork head	15
Basic region leucine zipper & bZIP	6
Helix-loop-helix DNA binding domain	12
Myb-like DNA-binding domain	3
Zinc finger C2H2 type	3
Zinc finger MIZ type	1
HMG box	12
TBOX	5
ETS domain	12
MADS domain	4

### Annotation of *Symbiodinium-*derived contigs

Holobiont coral tissues also contain eukaryotic dinoflagellate endosymbionts of the genus *Symbiodinium*[30,31]. We therefore determined the contribution of symbiont-derived transcripts in our analysis. First, we extracted the regions of cDNA contigs that corresponded to each individual annotated ORF in two datasets (For commands, see Additional file 1: File S1). Furthermore their similarity search against two *Symbiodinium* transcriptomes (http://medinalab.org/zoox/) was performed using BlastN. In order to define an E-value as a cutoff threshold, a reciprocal BlastN search between the *N. vectensis* genome and the two *Symbiodinium* transcriptomes showed an average E-value of e^-80^. Thus all contigs with similarity higher than this threshold to *Symbiodinium* were defined as likely to be symbiont-derived. Based on these results, 9% of the annotated ORFs (1.34% of the total assembled contigs) of *Fav1* were labeled as symbiont sequences, and 8.7% (1.61% of total assembled contigs) of *Fav2*. FASTA files of these non-symbiont transcripts are reported (Additional file 11: File S9, Additional file 12: File S10). Finally, we performed BlastX (E-value equal to at least e^-30^) on the non-symbiont derived cDNA fragments against the *N. vectensis* proteome to confirm correct initial annotation by BlastP. All the cDNA sequences matched to the same *N.vectensis* IDs that were predicted using BlastP.

### Phylogenetic assessment

Molecular markers are essential tools for population genetic studies. Typically, combination of mitochondrial and nuclear markers are used to examine the species relationships. In order to generate a *Favia* molecular marker dataset, we downloaded *Favia* related sequences from NCBI. Similarity searches were carried out against this *Favia* dataset. Among various molecular markers, we chose COI, Cytb and 28S. Individual sequence regions were identified and extracted from the cDNA contig files in both samples. DNA alignments for each locus were generated using ClustalW2 with default parameters [32] (Additional file 13: File S11, Additional file 14: File S12, Additional file 15: File S13). Consequently, a matrix of these three loci was generated using FASconCAT [33]. A Maximum likelihood phylogenetic analysis (RaxML) was carried out [34]. Maximum likelihood phylogenetic analysis using three loci (COI, Cytb, 28S) suggests that these *Favia* samples belong to *Faviids* (Additional file 16: Figure S2)*.* Morphological analysis places them as *F. albidus*[17]*,* a species that is not yet represented in NCBI. For example, out of 18 *Favia* species that have been described morphologically, only 15 of them have molecular data in NCBI. *F. albidus*, *F. helianthoides*, and *F. marshae* lack molecular markers in NCBI. Based on geological distribution [17] and morphology, we suggest these two species belong to *F. albidus*. In fact, *F*. *helianthoides* has no morphological similarities with our samples, and *F. marshae* habitat has never been reported in Red Sea [17]. However, further skeletal samplings are required for final validation [35,36]. Regardless, this study increases the protein information of the *Faviids* from 496 proteins to over 12,000 proteins in NCBI.

### Characterization of one exemplary homologous protein cluster

**Figure 3:**
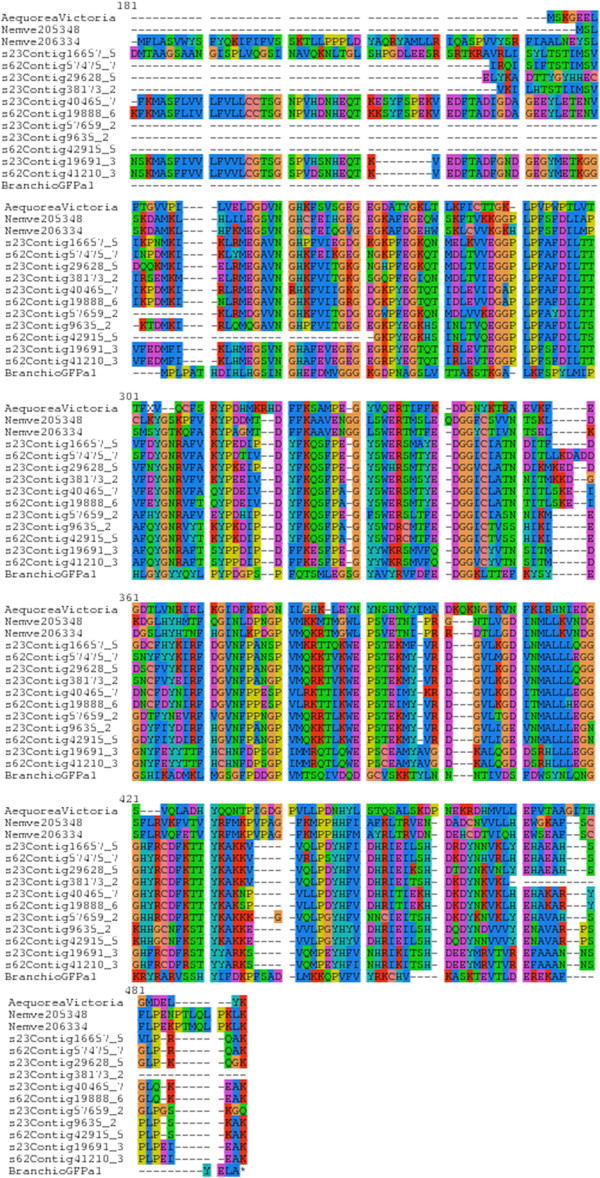
**Overlapping region of amino acid sequence alignment of one exemplary cluster of identified homologous protein clusters.** This gene family belongs to naturally expressed fluorescent protein. Conserved chromophore region (XYG) is located at the position 303–305. The newly identified sequences with extended N-terminal are s23Contig16657-5, s23Contig40465-7 in *Fav1*, s62Contig19888-6, and s62Contig41210-3 in *Fav2*. The full-length alignment is reported in Additional file 17: Figure S3.

From the protein clustering results, we chose to characterize a protein family with a natural fluorescent property. One of the benefits of utilizing scleractinian corals as our model organism is that they posses genes for fluorescent proteins (FPs), a rare characteristic in most other Phyla’ besides Cnidaria [37-40]. In *N. vectensis*, six protein IDs encode for FPs [41]. A search among the homologous sequence clusters with E values of at least 2e^-30^ in each transcriptome led to the identification of one protein cluster group per sample representing potential fluorescent proteins (FPs). A total of 11 new potential FPs were identified, six belonging to the *Fav1* sample and four belonging to the *Fav2* sample. One additional sequence, s23Contig9635-2 was found by increasing the E-value to 2e^-10^ in *Fav1*. The alignment of these sequences with *N. vectensis* fluorescent protein sequences (JGI ID:205348, ID:206334), *Branchiostoma GFPa1*[42] and GFP of *Aequorea victoria* (GI:17943301) showed a considerable homology (Figure 3). The conserved chromophore region is located at the residues 303 to 305 based on the top sequence. Our data shows that one of the newly identified potential fluorescent protein sequences (*Fav1* s23Contig16657-5) is 185 amino acids longer at the N-terminus (416 amino acids in total) and two of them were shown to be 49 (*Fav2* s62Contig19888-6) and 41 amino acids (*Fav2* s62Contig41210-3) longer than the consensus length of reported sequences in NCBI (wild-type GFP from *Aequorea victoria* is 236 amino acids) (Additional file 17: Figure S3). This extended region does not seem to interfere with the proper folding and expression of FP, however further studies are required to reveal the function of these upstream domains.

**Figure 4:**
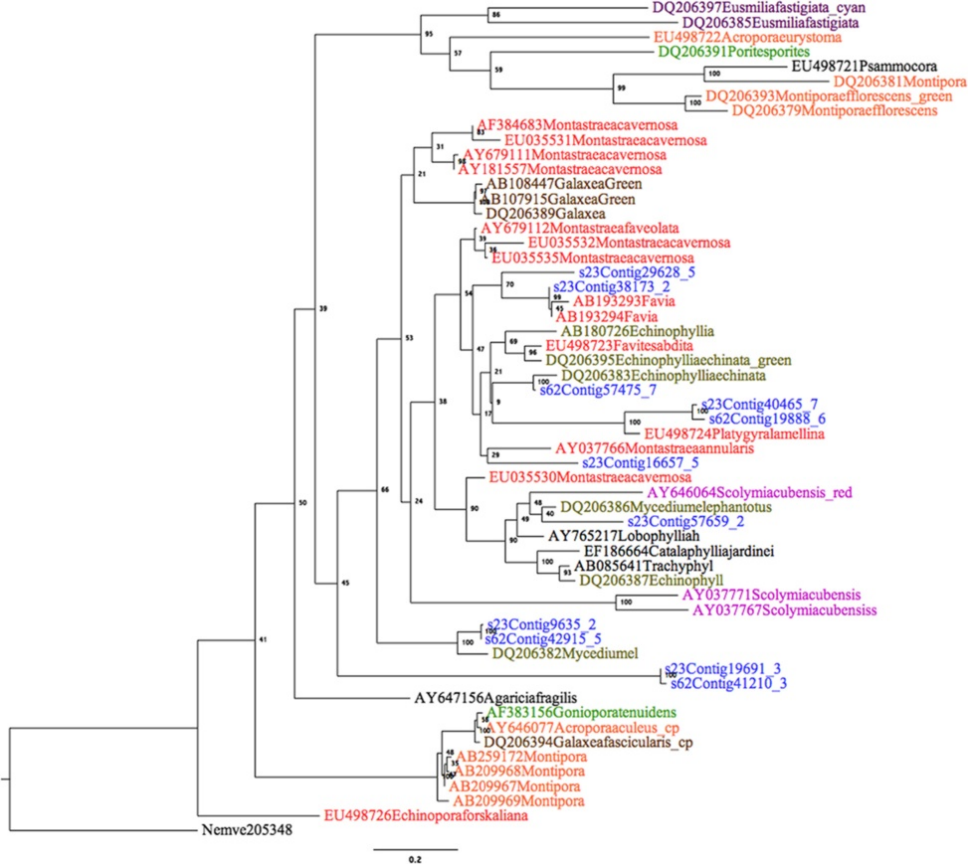
**Maximum likelihood tree of 46 known fluorescent proteins and 11 newly identified fluorescent protein sequences using RaxML.** The alignment was 1,000 times bootstrapped and one FP sequence from *N. vectensis* was the out-group. The newly identified FP sequences are colored blue. Other colors represent different coral families; Faviidae, red; Acroporidae, orange; Oculinidae, brown; Pectiniidae, dark green; Meandrinidae, dark purple; Mussidae, pink; Poritidae, green; Node labels are bootstrap supports. See Additional file 19: File S14 for information on alignment.

Furthermore, the maximum likelihood trees were generated from the alignment of 156 fluorescent sequences, including the 11 newly identified sequences (Additional file 18: Figure S4, Additional file 19: File S14 contains all the accession numbers). There was a strong bootstrap support for basal clade relationships within tree. This includes the order Ceriantipatharia, and Pennatulacea, although low bootstrap support for FPs within order Scleractinia*.* Ctenophore FP clustered with hydrozoan FP, therefore the cnidarian clade was not monophyletic. Others have shown that incongruence with taxonomy is not unusual in fluorescent proteins [43]. For better visualization, a smaller maximum likelihood sub-tree was generated from 46 scleractinian FP sequences (Figure 4). Although the bootstrap values improved compared to Additional file 18: Figure S4, some branches still exhibited low bootstrap values. Nonetheless, using RaXML [27], we categorized the newly identified sequences into four clades and using ProtTest [44] we identified “PROTGAMMAWAGF” as the best-fit model.

In order to evaluate our assembly method and the possible impact of ABySS-specific errors on the annotation accuracy of the long candidate FP sequence, we performed both Trans-ABySS [20] and Trinity [45] on reads from *Fav1*. Both assembly programs led to the generation of sequences identical to *Fav1* s23Contig16657-5 as predicted using ABySS and CAP3. (Additional file 20: File S15).

### Validation of the identified protein clusters as fluorescent proteins

**Figure 5:**
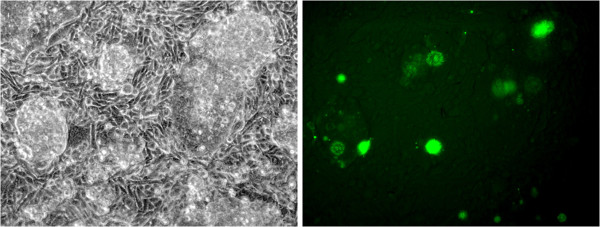
**Expression of an assembled contig in HEK293 mammalian cells yields fluorescence.** An open reading frame of contig 19888 from *Fav2* was synthesized using mammalian preferred codon usage (887 bases of s62Contig19888) and subcloned into pcDNA 3.1, and transfected into HEK293 mammalian cells using Fugene (Boehringer-Mannheim). The left panel depicts a phase contrast image of transfected HEK293 cells, and the right panel depicts fluorescence (using FITC excitation and emission) from the same field. Scale bar = 100 microns.

The intrinsic fluorescence of FPs includes a unique chromophore that is formed post-translationally within the protein upon autocatalytic cyclization and oxidation of residues X-Tyr-Gly [46]. The fluorophore is located almost at the center of the cylinder and is inaccessible to outside enzymes [46,47]. The GFP fluorophore is capable of forming under a wide range of conditions and once formed is highly stable. The entire structure is very resistant to denaturation by heat and denaturants. The three sequences with longer N-terminal domains (s23Contig16657-5, s62Contig19888-6 and s62Contig41210-3) were cloned into mammalian expression vectors. We used Kozak analysis [48] to pick the best potential start codon, and reading frames were generated using gene synthesis. The start codons are underlined in red in Additional file 21: Figure S5. The synthesized sequences were optimized for expression in mammalian cell lines. The synthesized sequences showed fluorescence when expressed in HEK-293 mammalian cells, thus validating them as genuine FPs (Figure 5).

### *In Silico* quantification of *Faviids* transcripts

In order to rule out the possibility of promiscuous domain assembly, we assessed the quality of the *de novo* assembly of FP sequences, as well as all other transcripts, by mapping reads on assembled contigs for each sample. Such read alignment to contigs is necessary to provide support for new transcript identification as well as for determining gene expression levels [49,50]. In order to measure the Reads Per Kilobase of exon model per Million mapped reads (RPKM) [50], a sub-fasta cDNA region, corresponding to each ORF, within each contig was generated. Reads were aligned to these annotated cDNA regions. Coverage (RPKM) measurements were determined using a Perl script (Script 3). The results are reported (Additional file 22: File S16, Additional file 23: File S17). The mapping of all the reads onto the annotated *Faviids* transcript showed that the number of reads corresponding to each transcript ranged from 10 to 47,189, with an average of 850 reads per transcript in *Fav1*, and 10 to 29,222, with an average of 766.37 reads per transcript in *Fav2*, indicating a wide range of expression level of *Faviids* transcripts. It also indicates that very low expressed annotated *Faviids* transcripts were also represented in our assembly. The minimum coverage (RPKM) of an annotated *Fav1* transcript was 3.89 and maximum of 6,919.20 with an average of 68.61. The RPKM ranged from 3.60 to 8,576, with an average of 72.64 in *Fav2.* The average and the range of RPKM per transcript is similar and somewhat higher (25.7) than other whole transcriptome studies [26].

**Figure 6:**
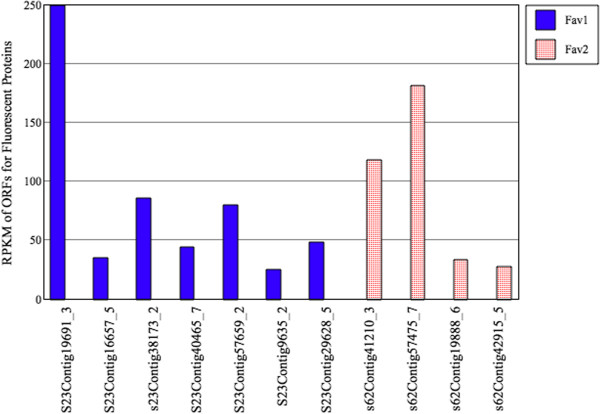
***In silico *coverage plot of the read-to-contig alignment measurements.** The cDNA fragments with annotation for fluorescent protein coverage measurements.

All the cDNA regions annotated for fluorescent property had reasonable coverage, including the long candidate cDNA sequence (*Fav1* s23Contig16657-5) (Additional file 21: Figure S5). Based on the calculated RPKMs for each of the identified fluorescent protein in both samples*,* s23Contig19691-3 in *Fav1*, and s62Contig57475-7 in *Fav2* had the highest coverage level (Figure 6).

## Conclusions

In this study, we demonstrate a gene clustering strategy and utilize this in conjunction with NGS contig assembly, sequence conservation measurements, annotation and expression quantification for *de novo* assembled transcriptomic data. Working with two uncharacterized Faviid corals, we report 120,000 non-redundant transcripts to a genus whose sequence data was previously limited to 496 in public databases. These results provide greatly enhanced access to the expressed genes in Faviidae reef building corals, a potentially valuable resource of genetic/functional markers for population structure and functional genomic studies. We also took advantage of the optical properties of these corals expressed fluorescent proteins to validate our annotation methods to show that these sequences were indeed *bonafide* fluorescent protein genes. These methods reported in this study are available via Open Source software programs as well as our provided scripts.

## Methods

### Coral collection and total RNA isolation

This study was conducted during the period of May–June 2009 on a coral reef on the northern tip of the Gulf of Eilat, in the northern Red Sea (29º30′N, 34°55′E).

Samples were collected at 65 m, using closed-circuit trimix rebreather system (Megalodon™). The organisms were identified under water to the family level, *Faviidae*, and brought to the surface in a black mesh bag to avoid sun exposure. The organisms were immediately photographed and vouchered with white light and fluorescent photography as described in [51] and stored in a shaded running-seawater facility. Within 1–2 hours of collection, samples were rinsed in sterile-filtered artificial seawater and processed for RNA and DNA. The tissue of the coral was extracted from the skeleton using QiaShredder (Qiagen). For RNA, the TriZol method was used and stored as an ethanol precipitate for travel back to the US. DNA was extracted using Qiagen DNAeasy kit according to manufacturer’s protocol and stored in at 4°C. The specimens have been photo vouchered and their genomic and transcriptomic raw materials are stored in the American Museum of Natural History Ambrose Monell Cryo Collection.

### Preparation and screening of cDNA library

Illumina sequencing using the GAII platform was performed at the Yale University W.M. Keck Biotechnology Resource Laboratory according to manufacturer’s instructions (Illumina, San Diego, CA) (Additional file 24: File S18) and using high quality RNA with a 28S rRNA band at 4.5 kb that is at least twice the intensity of the 18 s rRNA band at 1.9 kb. The cDNA library contained 77,804,306, 75-mer length reads. The sequencing data are deposited in NCBI Sequencing Read Archive [52]. (The BiosampleIDs = SAMN01761696, SAMN01761695).

### *De novo* assembly

*De novo* assembly was carried out using ABySS with default settings across multiple k-mer values [18]. After assessing different k-mer values, the three best k-mer assemblies (35-mer, 39-mer, 45-mer for *Fav1* and 31-mer, 35-mer, 39-mer for *Fav2*) were selected and concatenated for the second step of assembly. To evaluate the N50 length and the number of assembled contigs using different k-mer values, we used a Perl script. CAP3 [22] was used to remove redundancy across ABySS assemblies and to merge contigs into longer sequences. All assembled contigs were subjected to annotation and further protein homology searches. Trans-ABySS [20] and Trinity [45] were used to confirm the long ORFs, homologous to fluorescent proteins, which were identified with ABySS and CAP3.

### Gene annotation and analysis

A set of possible Open Reading Frames (ORF), stop to stop from assembled sequences, was generated using EMBOSS [24]. To annotate the *de novo* assembled sequences, a similarity search against *N. vectensis* proteome was conducted using BLASTP with two E values of 2e^-10^ and 2e^-30^. The resulting data (E-value of 2e^-30^) was filtered and clustered using TRIBE-MCL [27]. Each homologous group was annotated using GO and KOG annotated *N. vectensis* data (http://genome.jgi-psf.org/Nemve1/Nemve1.download.html). For *Symbiodinium* peptide annotation, a homology search using BLASTN with E values of 2e^-80^ against the *Symbiodinium* transcriptome (http://medinalab.org/zoox/) was carried out. The final non-symbiont FASTA cDNA fragments were reported.

### Completeness measurement

The BlastP (E-value of 2e^-30^) output list generated from homology search of both samples against *N. vectensis*[41] and *A. digitifera*[53] was organized for completeness measurements. The completeness formula according to [28] was implemented into a Perl script (*In prep*) to determine the percentage of the reference proteome that is covered by each of our sets of assembled transcripts. Length coverage of each of these reference ORFs by a hit from our data set had to be at least 80%.

### Phylogenetic analysis of FPs

The maximum likelihood tree of identified fluorescent protein was generated using RaXML [34] under PROTGAMMAWAGF amino acid substitution model, selected based on the results from ProtTest [44]. The alignment was generated using MAFFT [54] and CLUSTALW2 [32] with minor adjustment at the N-terminus region, when long gaps were inconsistent with other isoforms. Bootstrap values were estimated based on 1,000 replicates and were given for all presented branches. The variant sites were visualized with geneious (http://www.geneious.com). Dendroscope was used for visualization [55].

### Phylogenetic assessment

Molecular barcodes for all the *Favia* related sequences were downloaded from NCBI. A similarity search with sequences from our annotation was carried out against this *Favia* dataset. Cytb, COI and 28S sequences were identified and extracted from the cDNA contig files in both samples. DNA alignments for each locus were generated using ClustalW2 with default parameters [32]. Consequently, a matrix of these three loci was generated using FASconCAT [33]. A Maximum likelihood phylogenetic analysis (RaxML) was carried out [34]. Bootstrap values were estimated based on 10,000 replicates and were given for all presented branches. Dendroscope was used for visualization [55].

### Cloning of fluorescent proteins

The three cDNA sequences (*Fav1* s23Contig16657, *Fav2* s62Contig19888-6 and *Fav2* s62Contig41210-3) were synthesized and propagated in pUC57 (GenScript USA Inc.). Kozak [48] analysis was used to determine the location of the potential start codon. The genes were subcloned from pUC57 into the NotI-BamHI site of the mammalian expression vector pcDNA 3.1 (Invitrogen, Inc.) using standard recombinant techniques [56].

### *In Silico* gene coverage measurements

Gene coverage levels were determined using a Perl script (Script 3). This script implements Bowtie [57] to map reads to an annotated reference cDNA, and calculates the RPKM according the formula used in [50]. For visualization, BWA [58] was used to generate the read-to-contig alignment. The annotated cDNA from individual samples were used as the reference contig, and SAMtools [59] was used to generate binary files to be visualized in the IGV [60] genome viewer (For commands, see Additional file 1: File S1).

## Competing interests

The authors declare that they have no competing interests.

## Authors’ contributions

DFG, RD, VAP and SFPM designed the study. SFPM carried out the molecular genetic studies. SFPM and AN designed the bioinformatics pipeline and data handling scripts. RD coordinated the statistical analysis. H-T K, VAP and DFG designed synthetic genes for expression in mammalian cells. ZH, VAP, DT and DFG participated in sample collection and Illumina sequencing. DFG, RD, VAP and SFPM drafted the manuscript. All authors read and approved the final manuscript.

## Supplementary Material

Additional file 1: File S1 Parameters and commands used in this manuscript. Click here for file

Additional file 2: Files S2 BlastP parsed output files against *N. vectensis* proteome for sample *Fav1* and *Fav2* with 2e-30. Click here for file

Additional file 3: Files S3 BlastP parsed output files against *N. vectensis* proteome for sample *Fav1* and *Fav2* with 2e-30. Click here for file

Additional file 4: Files S4 TRIBE-MCL input files. Click here for file

Additional file 5: File S5 TRIBE-MCL input files. Click here for file

Additional file 6: Files S6 Homologous protein clusters (TRIBE-MCL) output for sample *Fav1* and *Fav2.* Click here for file

Additional file 7: File S7 Homologous protein clusters (TRIBE-MCL) output for sample *Fav1* and *Fav2.* Click here for file

Additional file 8: Table S1 Completeness metrics for two samples compared to *N. ventensis* and *A. digitifera.* Click here for file

Additional file 9: Files S8 GO,KOG, InterPro annotation for homologous protein clusters in *Fav1.* Click here for file

Additional file 10: Figure S1 Distribution of *Fav1* transcript clusters in different GO categories. Click here for file

Additional file 11: Files S9 FASTA files for cDNA region encoding for non-symbiont annotated ORFs in *Fav1* and *Fav2.* Click here for file

Additional file 12: File S10 FASTA files for cDNA region encoding for non-symbiont annotated ORFs in *Fav1* and *Fav2*. Click here for file

Additional file 13: File S11 Alignment of *Fav1* and *Fav2* Cytb nucleotide sequences, including other *Favia* species. Click here for file

Additional file 14: File S12 Alignment of *Fav1* and *Fav2* COI nucleotide sequences, including other *Favia* species. Click here for file

Additional file 15: File S13 Alignment of *Fav1* and *Fav2* 28S nucleotide sequences, including other *Favia* species. Click here for file

Additional file 16: Figure S2 Maximum likelihood tree of three loci (COI, Cytb, 28S). Data matrix was generated from 15 *Favia* species and *Fav1* and *Fav2*. Nucleotide sequences were aligned using clustalw2 with default parameters, the 3 loci matrix was generated using FASconCAT, and the tree was constructed using RaxML (See methods). *Montastrea cavernosa* is selected as the out-group. Click here for file

Additional file 17: Figure S3 Amino acid sequence alignment of full-length fluorescent protein isoforms. Click here for file

Additional file 18: Figure S4 Maximum likelihood tree of 156 known fluorescent proteins, including our 11 newly identified sequences using RaxML. Shows the relationships of the major groups of known fluorescent proteins. Major lineages cluster together, although Ctenophore and Hydrozoa do not form a monophyletic group. Within Anthozoa class, order Ceriantharia (orange); Actinaria (red); Pennatulacea (dark green); and Scleractinia (black); Hydrozoa (purple); Copepoda (light green); Ctenophora (blue); Chordata (turquoise blue), most basal group; Newly identified sequences are colored blue within Scleractinia. The alignment was 1,000 times bootstrapped and *B. floridae* was the out-group. Click here for file

Additional file 19: File S14 Alignment of 156 known fluorescent proteins, including the 11 newly identified FP sequences. Click here for file

Additional file 20: File S15 Search result in Trans-ABySS and Trinity assembly output for homologous contig, similar to identified *Fav1* s23Coting16657-5 produced by ABySS and CAP3. Click here for file

Additional file 21: Figure S5 Read-to-contig alignment. 75 bp read alignments to the coding region of s23Contig16657-5, 1,377 bp total length. Click here for file

Additional file 22: Files S16 RPKM measurement for all annotated cDNA regions from *Fav1* and *Fav2*. Click here for file

Additional file 23: File S17 RPKM measurement for all annotated cDNA regions from *Fav1* and *Fav2*. Click here for file

Additional file 24: File S18 Protocol for preparing samples for sequencing of mRNA.
**Scripts:** Script 1: Perl script for performing blast search. Script 2: Perl script for pre-clustering the blast parsed file. Script 3: Perl script to calculate RPKM for the assembled file. Script S1: Perl script to shuffle short read sequences. Script S2: Perl script to measure the N50 statistics. Script S3: Unix shell script to remove Fasta files shorter than a threshold. Script S4: Generate the sub-Fasta file. Script S5: Extract the cDNA sequences corresponding to ORF files. Click here for file
