# Supplementary material for: Transcriptome deep-sequencing and clustering of expressed isoforms from Favia corals
Source: BMC Genomics. 2013 Aug 12;14:546. doi: 10.1186/1471-2164-14-546 (PMC3751062; doi:10.1186/1471-2164-14-546)
Supplement: Additional file 1: File S1 — Parameters and commands used in this manuscript. [file 1471-2164-14-546-S1.docx]

Supplementary file S1.

For the optimal execution of the following bioinformatics workflow, a Unix-type operating system is required. The assembly of datasets requires the use of 64-bit Linux operating system with at least 1GB of RAM per million reads.

*All customized scripts used here are available at: https://github.com/spooyaei/coralAssembly

*****The s23 and s62 contig prefix labels in the supplementary files represent *Fav1* and *Fav2*, respectively.

**1-Assembly**

Individual paired-end fastq reads were shuffled and assembled using the short read assembly program ABySS (1.3.3) to generate multiple k-mer sequences. Statistical evaluation (N50 values) was performed on each k-mer assembly.

(a) Requirements: Software installation. A version of ABySS can be found at: <http://www.bcgsc.ca/platform/bioinfo/software/abyss/releases/1.3.0>;

Scripts:

“**ShuffleSequences_fastq.pl”** (Perl Script to shuffle the reads; Script S1)

**“amstat.pl”** (Perl script for statistics; Script S2)

**“ rmshort_contigs.sh”** (Unix shell script to remove shorter than certain length; Script S3)

(b) Input files: Read1.fastq Read2.fastaq

(c) Commands:

- shuffleSequences_fastq.pl Read1.fastq Read2.fastq combined.fastq
- abyss-pe np=8 k=(variable) n=10 q=3 v=-v name=seq1 in=combined.fastq

Output files: seq1-bubble.fa; seq1-indel.fa; seq1-contig.fa; seq1.path1;seq1.path2; seq1.adj

Note: The k-mer values could vary between read-length/2 up to read-length-1

- ./amstat.pl seq-contig.fa > seq-contigstat

Note: In order to save computation time for the next step but still retain the informative contigs, all contigs shorter than 150nt in two of the k-mer assemblies were removed. All the contigs in one of the k-mer assemblies were kept.

- rmshort.contigs.sh seq1-contig.fa > seq1-withlong150contig.fa

Note: Each seq-contig.fa should be assigned unique sequence identifier representing the k-mer values.

- cat seq1-withlong150contig.fa seq2-withlong150contig.fa seq3-contig.fa > all-kmers.fa

**2-Redundant sequence removal and length increment**

Remove redundant ABySS output contigs.

(a) Requirements: Software installation. A version of CAP3 can be found at: <http://seq.cs.iastate.edu/>.

(b) Input files: all-kmers.fa

(C) Commands:

- Cap3 all-kmers.fa > all-kmer.out

Output files: all-kmer.fa.cap.ace;all-kmer.fa.cap.contigs ; all-kmer.fa.cap.links; all-kmer.fa.cap.qual; all-kmer.fa.cap.info; all-kmer.fa.cap.singlets; all-kmer.out

- ./amstat.pl all-kmer.fa.cap.contigs > all-kemr.capstat

**3- ORF prediction.** Six-frame open reading frames predicted for each of the assembled contigs.

(a) Requirements: Software installation. A version of EMBOSS, getorf can be found at:

<http://emboss.sourceforge.net/apps/cvs/emboss/apps/getorf.html>

Note: We used this program to generate the six-frame ORFs from stop to stop with a minimum size of 90AA.

(b) Input files: all-kmer.fa.cap.contigs

(C) Commands:

- ./getorf -sequence all-kmer.fa.cap.contigs -outseq all-kmer.fa.cap.contigs.orf90.fas -minsize 90 -find 0 -table 0 -reverse Y

**4-Similarity search:** The predicted ORFs were compared to the *Nematostella vectensis* and *Acropora digitifera* proteomes using BlastP and BlastX (embedded in custom-built Perl scripts). For each species, the query sequences with the top bitscore were identified (Files S4, S5).

(a) Requirements: Software installation. A version of EMBOSS,getorf can be found at:

<http://blast.ncbi.nlm.nih.gov/Blast.cgi?CMD=Web&PAGE_TYPE=BlastDocs&DOC_TYPE=Download>.

The *N.vectensis* proteome can be found at :

<http://genome.jgi-psf.org/Nemve1/Nemve1.home.html>

The *A.digitifera* proteome can be found at :

<http://marinegenomics.oist.jp/genomes/gallery>

Scripts:

**“ blast-forked.pl”** ( Perl Script to run Blast; Script 1)

**“ tophit.pl” (**Perl script to generate Tribe-MCL input from blast parsed file; Script 2 )

**“completeness.pl” (**Perl Script to measure completeness; In Prep)

(b) Input files: all-kmer.fa.cap.contigs.orf90.fas; Nematostella.prot.fasta; Acropora_digitifera.prot.fas

(c) Commands:

- formatdb -i contig.fas –p T-o T -V &
- ./blast_forked.pl --blastdb Nem.fas --fasta sam23.orf90.fas --config config.e30.blastp --outdir sam23.nemblastp.e30 --procs 24

Note: We used BlastP to search against *N.vectensis* and *A.digitifera* for completeness measurements.

Output: topbitscrore.blastparsed.txt (Supplementary Files S4, S5). This is the input for Tribe-MCL.

**5- Cluster the ORFs around top Blast hit from *N. vectensis*.** The similar protein sequences from *Fav1* and *Fav2* were clustered around individual protein IDs from the reference proteome.

(a) Requirements: Software installation. A version of TribeMCL can be found at:

<http://micans.org/mcl/>

(b) Input files: topbitscrore.blastparsed.txt (Files S4, S5)

(c) Commands:

mcl cluster.list.txt --abc -o cluster.list.txt.out

(d) Outputs: clusterlist.txt.out (files S6, S7)

**6- Functional annotation of putative protein clusters.** Each domain (or isoform) cluster group was functionally annotated using GO, InterPro, and KOG annotaton files from *N. vectensis*.

(a) Requirements: Micorosoft Excel 2010; *N.vectensis* GO, InterPro, and KOG files (available at: <http://genome.jgi-psf.org/Nemve1/Nemve1.download.ftp.html>)

(b) Input files: *Fav1* cluster.list.txt.out (File S6)

(C) Commands: The Vlookup command in Excel was used to search the GO and InterPro domain annotation files for *N. vectensis* protein IDs. If an entry did not have a GO ID, InterPro, or KOG match, it was labelled “NA.”

Outputs: *Fav1* functional annotation; File S8.GOannotation (File S8)

**7- Symbiont annotation.**

(a) Requirements:

Scripts:

**“ blast-forked.pl”** ( Script 1)

**“ getsubfasta.pl”** ( Perl script to generate subfasta file having the id list; Script S4)

**“extractcdna.pl”** ( Perl script to extract cDNA nucleotide sequences using the ORF Amino Acid coordinates; Script S5)

**- Generate nucleotide cDNA sub-fasta sequences from Tribe-MCL output IDs**

- awk '{print >("orthlist_" int((NR+1)/1))}' combined.tribe.out

Output: 9398 id files (*Fav1* and *Fav2* combined)

- for i in orthlist_*; do sed 's/\t/\n/g' $i > n$i; done

Output: formatted id files

- for i in northlist_*; do perl getsubfasta.pl $i > $i.fas; echo $i; done

Note: the $i is the id for all the isoform clusters. “getsubfasta.pl” uses those ids to extract the annotated ORFs from original ORF files.

Output: Homologous ORF fasta sequences.

- cat fav1.cap.fas fav2.cap.fas > fav1.fav2.cap.fas
- ./extract-cdna.pl –a genfam.fav1.fav2.orf.fas –n fav1.fav2.cap.fas > genfam.fav1.fav2.cdna.fas

Output: The cDNA nucleotide fasta sequences from *Fav1* and *Fav2* that are homologous to *N.vectensis* preoteins.

**- Symbiont annotation**: cDNA sequences of the homologous isoform clusters with high levels of similary to symbiont transcriptome sequences were identified. In order to define the cutoff E-value for BlastN, a reciprocal BlastN search between the *N.vectensis* genome and two symbiodinium transcriptomes was performed. The average E-value for matches was 2e -80. Therefore, we used it as the cutoff.

- cat mf105_assembly.fasta kb8_assembly.fasta > symbiodata

Note: Symbiont database is generated using the symbiont transcriptome.

- ./blast_forked.pl --blastdb symbiodata.fas --fasta genfam.fav1.fav2.cdna.fas --config blastn.e30.config --outdir genefam.cdna.nem.blsn30 --procs 24

Output: list of cDNAs with match and without match to symbiodinium data.

- awk '$3 ~/No/' genefamilies.nuc.fas.symbiodata.fas.out.parse > genesthatarenotsymbio
- awk '$3 !~/No/' genefamilies.nuc.fas.symbiodata.fas.out.parse > genesthataresymbio
- less genesthatarenotsymbio | awk '{print $1}' > nonsymbiolist
- grep "fav1" nonsymbiolist| sort -u >fav1nonsymbiolist
- grep”fav2” nonsymbiolist| sort –u > fav2nonsymbiolist
- ./getsubfasta.pl fav1nonsymbiolist > genefam.nonsym.fav1.fas

Output: The annotated cDNA files in sample fav1 without symbiont (File S9)

- ./getsubfasta.pl fav2nonsymbiolist > genefam.nonsym.fav2.fas

Output: The annotated cDNA files in sample fav2without symbiont (File S10)

**8- *In silico* coverage measurement.** The individual annotated cDNAs were used as a reference for coverage measurements.

(a) Requirements: Software installation. Bowtie, BWA, and IGV can be found at:

<http://bio-bwa.sourceforge.net/>; <http://bowtie-bio.sourceforge.net/index.shtml>; <http://www.broadinstitute.org/igv/v1.3>

Scripts:

**“Bowtie-runner.pl”** (A customized perl script to measure coverage per contig; here, the contig is the annotated cDNA; Script 3)

(b) Input files: genefam.fav1.cDNA.fas; genefam.fav2.cDNA.fas; Reads from *Fav1* and *Fav2*

(c) Commands: (repeated for *Fav1* and *Fav2*)

- bowtie-build genfam.fav1.cDNA.fas fav1.cdnafasindex
- ./bowtie_runner.pl -s fav1.fastq -r genfam.fav1.cDNA.fas -c config.bowtie.best.a.y -i index.3 -o rpk.fav1cdna

Output: (File S16, S17)

**For visualizing the alignment in IGV:**

- bwa index -a bwtsw
- bwa aln genfam.fav1.cDNA.fas s_7_1_sequence.txt > file1.fav1.sai
- bwa aln genfam.fav1.cDNA.fas s_7_2_sequence.txt > file2.fav2.sai
- bwa sampe genfam.fav1.cDNA.fas file1.fav1.sai file2.fav1.sai s_7_1_sequence.txt s_7_2_sequence.txt |gzip > ga.fav1.gz
- samtools faidx genfam.fav1.cDNA.fas ( Output: *.fai)
- samtools import genfam.fav1.cDNA.fas.fai ga.fav1.gz fav1.cdna.bam
- samtools sort fav1.cdna.bam fav1.cdna.bam.sorted
- samtools index fav1.cdna.bam.sorted ( Output: *.bai, which is the input for IGV) (Figure S4)

**9- Species level clarification, using a three-locus phylogeny.**

(a) Requirements: Software installation. RaxML, Fascancat, and Geneious can be found at:

[http://software.zfmk.de](http://www.sciencedirect.com/science?_ob=RedirectURL&_method=externObjLink&_locator=url&_issn=10557903&_origin=article&_zone=art_page&_plusSign=%2B&_targetURL=http%253A%252F%252Fsoftware.zfmk.de) ; <http://www.lutzonilab.net/downloads/>; http://www.geneious.com/

(b) Input file: DNA Fasta files.

(c) Commands:

- ./blast_forked.pl --blastdb faviagene.fas --fasta fav1.cap.fas --config blastn.e10.config --outdir fav1.favia.blsn10 --procs 24

Output: cytb, COI, 28S sequences from *Fav1* and *Fav2*

- In Geneious, sequence similarity searches of the individual loci were carried out against the non-redundant (nr) database. For each locus, individual homologous sequences were aligned and saved as a .phy file (File S11, S12, S13)
- Fasconcat was used to build a matrix of all the loci
- RaxML: nohup raxml728 -T 8 -m GTRGAMMA -s coralbarcode.phy -o m.cavernos -n corbar8 -f a -x 12345 -N 10000

Output: A tree that is visualized using Dendropscope (Figure S2)
