# Supplementary figures and images for: Transcriptome deep-sequencing and clustering of expressed isoforms from Favia corals
Source: BMC Genomics. 2013 Aug 12;14:546. doi: 10.1186/1471-2164-14-546 (PMC3751062; doi:10.1186/1471-2164-14-546)

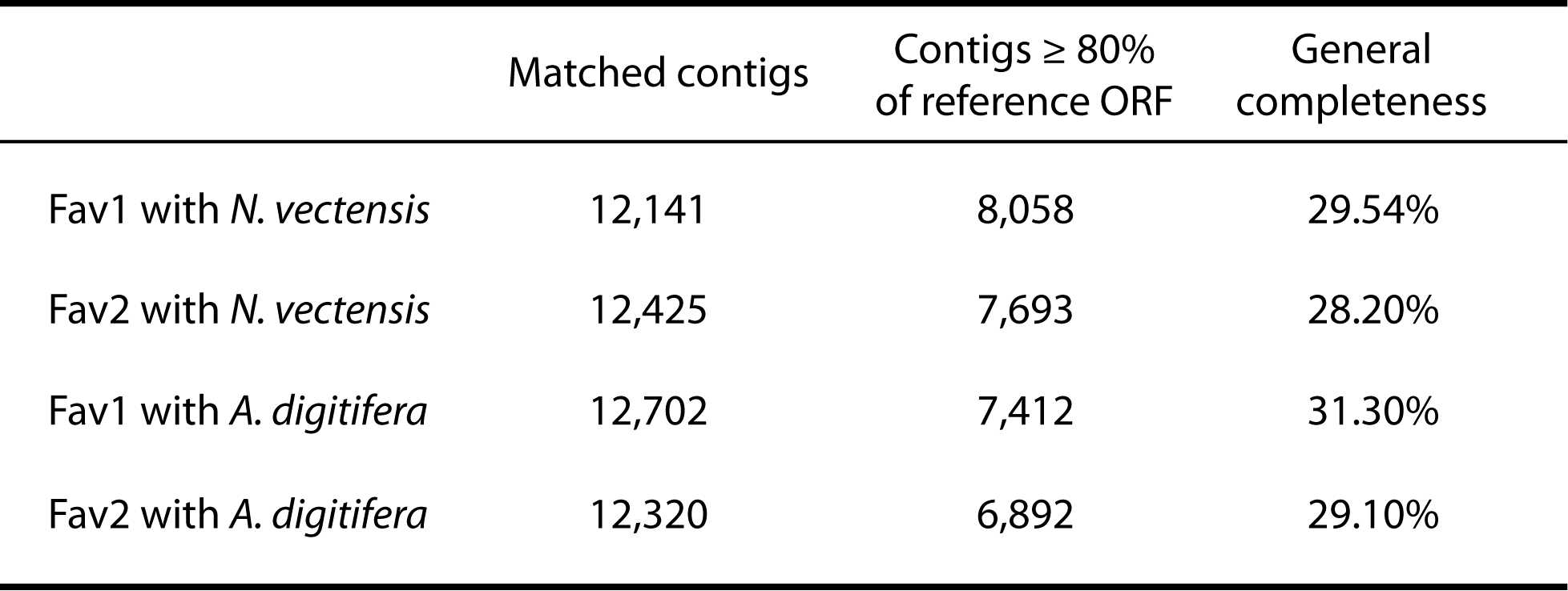

Supplement: Additional file 8: Table S1 — Completeness metrics for two samples compared to N. ventensis and A. digitifera. [file 1471-2164-14-546-S8.tiff]

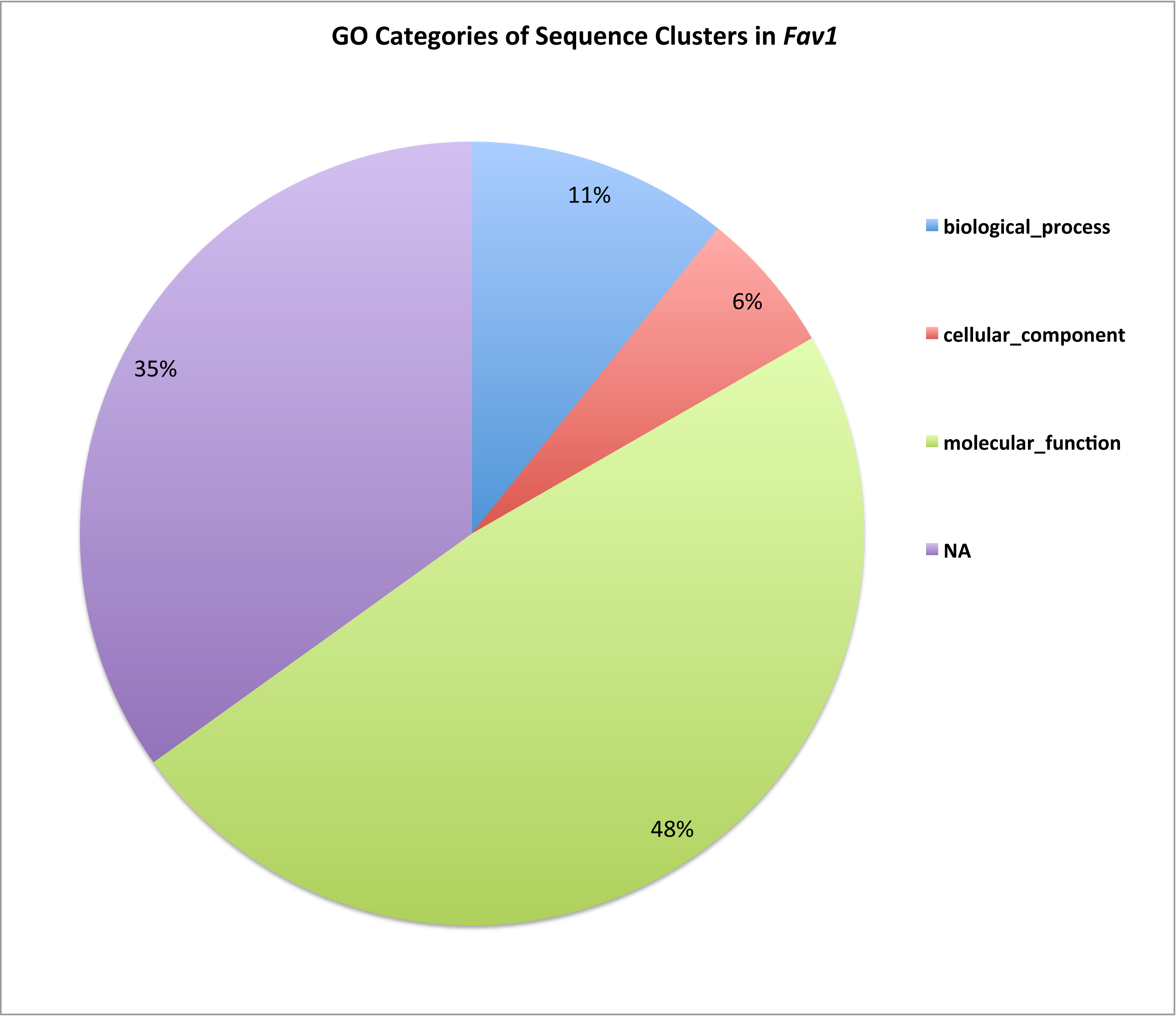

Supplement: Additional file 10: Figure S1 — Distribution of Fav1 transcript clusters in different GO categories. [file 1471-2164-14-546-S10.tiff]

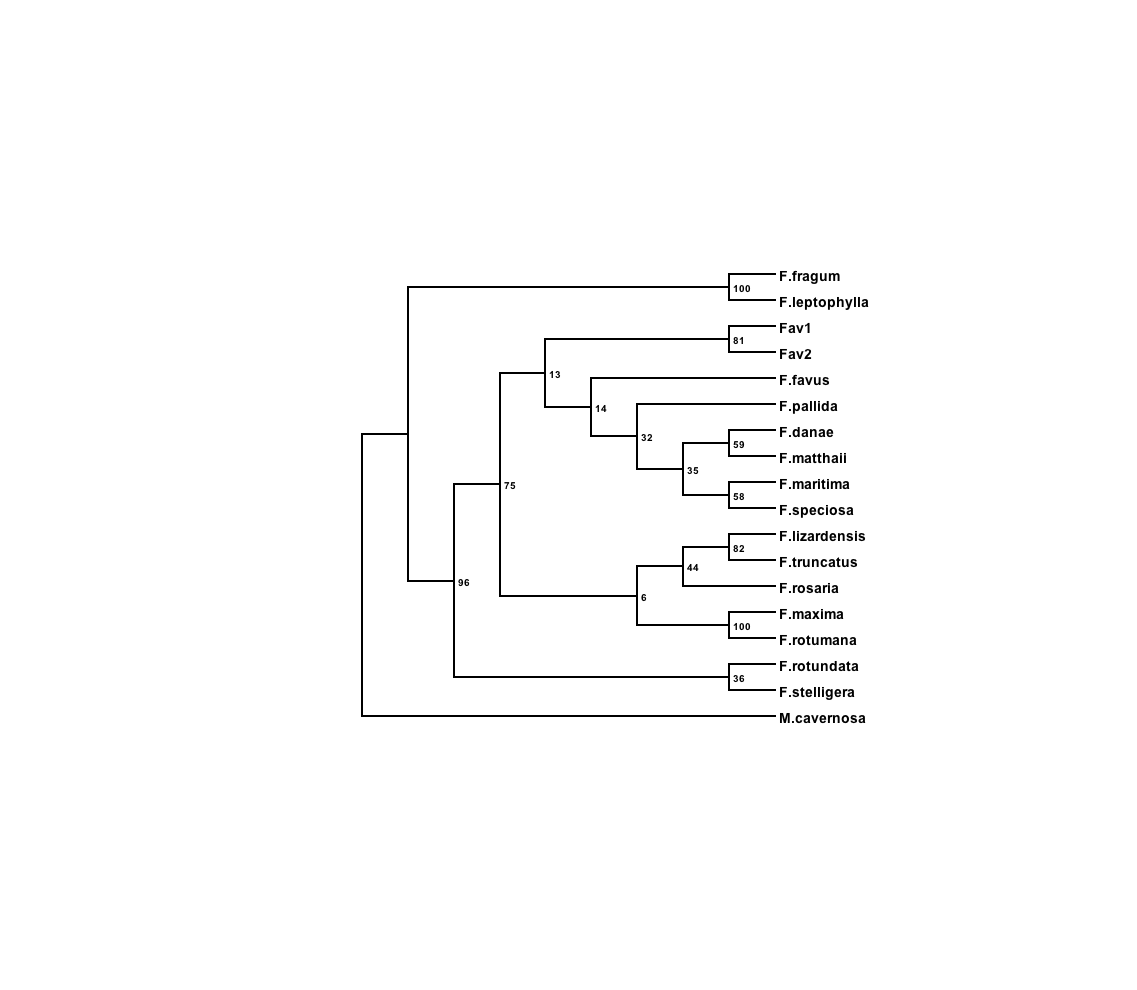

Supplement: Additional file 16: Figure S2 — Maximum likelihood tree of three loci (COI, Cytb, 28S). Data matrix was generated from 15 Favia species and Fav1 and Fav2. Nucleotide sequences were aligned using clustalw2 with default parameters, the 3 loci matrix was generated using FASconCAT, and the tree was constructed using RaxML (See methods). Montastrea cavernosa is selected as the out-group. [file 1471-2164-14-546-S16.tiff]

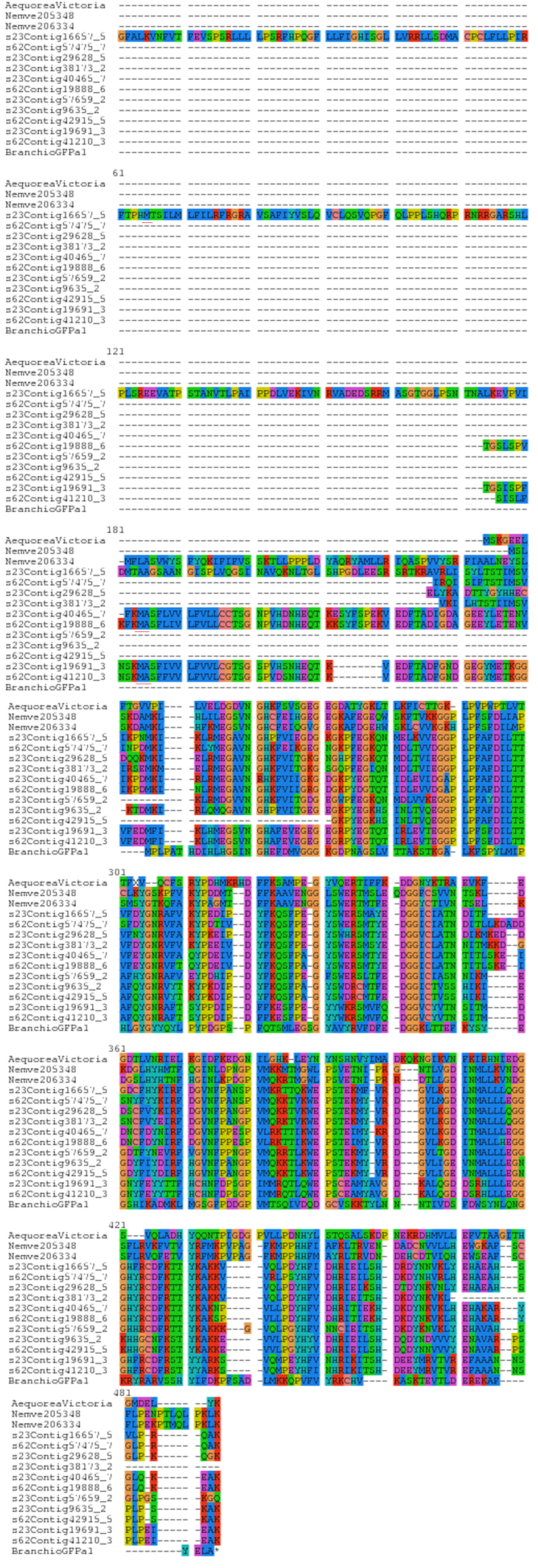

Supplement: Additional file 17: Figure S3 — Amino acid sequence alignment of full-length fluorescent protein isoforms. [file 1471-2164-14-546-S17.tiff]

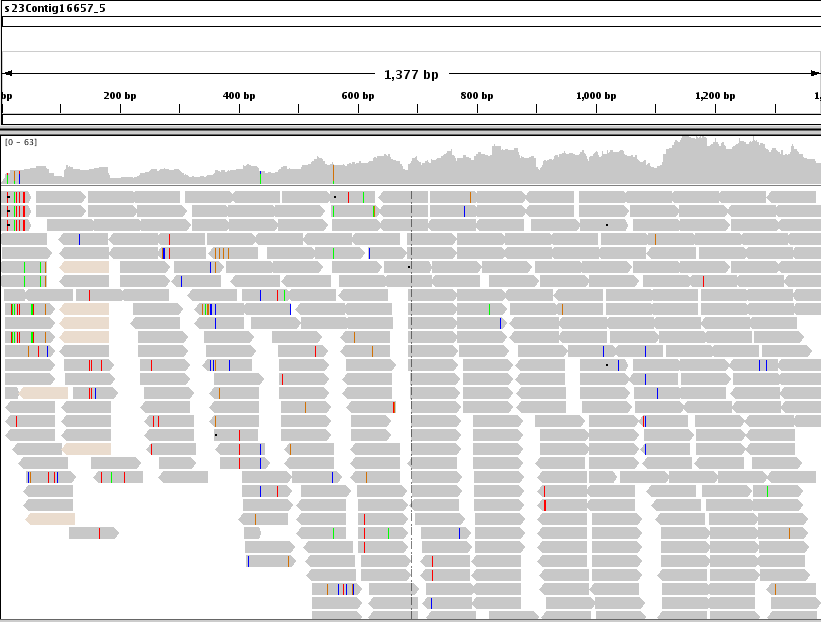

Supplement: Additional file 21: Figure S5 — Read-to-contig alignment. 75 bp read alignments to the coding region of s23Contig16657-5, 1,377 bp total length. [file 1471-2164-14-546-S21.tiff]
