# Supplementary material for: Transcriptome deep-sequencing and clustering of expressed isoforms from Favia corals
Source: BMC Genomics. 2013 Aug 12;14:546. doi: 10.1186/1471-2164-14-546 (PMC3751062; doi:10.1186/1471-2164-14-546)
Supplement: Additional file 24: File S18 — Protocol for preparing samples for sequencing of mRNA. Scripts: Script 1: Perl script for performing blast search. Script 2: Perl script for pre-clustering the blast parsed file. Script 3: Perl script to calculate RPKM for the assembled file. Script S1: Perl script to shuffle short read sequences. Script S2: Perl script to measure the N50 statistics. Script S3: Unix shell script to remove Fasta files shorter than a threshold. Script S4: Generate the sub-Fasta file. Script S5: Extract the cDNA sequences corresponding to ORF files. [file 1471-2164-14-546-S24.pdf]

Preparing Samples for Sequencing of mRNA

FOR RESEARCH ONLY

Topics

3 Introduction
4 Sample Preparation Kit Contents
7 Purify mRNA from Total RNA
9 Fragment the mRNA
10 Synthesize the First Strand cDNA
11 Synthesize the Second Strand cDNA
12 Perform End Repair
13 Add 'A' Bases to the 3' End of the DNA Fragments
14 Ligate Adapters to DNA Fragments
15 Purify cDNA Templates
17 Enrich the Purified cDNA Templates by PCR
18 Validate the Library

Part # 1004898 Rev. A
September 2008

illumina®

This publication and its contents are proprietary to Illumina, Inc., and are intended solely for the contractual use of its customers and for no other purpose than to operate the system described herein. This publication and its contents shall not be used or distributed for any other purpose and/or otherwise communicated, disclosed, or reproduced in any way whatsoever without the prior written consent of Illumina, Inc.

For the proper operation of this system and/or all parts thereof, the instructions in this guide must be strictly and explicitly followed by experienced personnel. All of the contents of this guide must be fully read and understood prior to operating the system or any of the parts thereof.

FAILURE TO COMPLETELY READ AND FULLY UNDERSTAND AND FOLLOW ALL OF THE CONTENTS OF THIS GUIDE PRIOR TO OPERATING THIS SYSTEM, OR PARTS THEREOF, MAY RESULT IN DAMAGE TO THE EQUIPMENT, OR PARTS THEREOF, AND INJURY TO ANY PERSONS OPERATING THE SAME.

Illumina, Inc. does not assume any liability arising out of the application or use of any products, component parts, or software described herein. Illumina, Inc. further does not convey any license under its patent, trademark, copyright, or common-law rights nor the similar rights of others. Illumina, Inc. further reserves the right to make any changes in any processes, products, or parts thereof, described herein without notice. While every effort has been made to make this guide as complete and accurate as possible as of the publication date, no warranty or fitness is implied, nor does Illumina accept any liability for damages resulting from the information contained in this guide.

© 2008 Illumina, Inc. All rights reserved. **Illumina**, **Solexa**, **Making Sense Out of Life**, **Oligator**, **Sentrix**, **GoldenGate**, **DASL**, **BeadArray**, **Array of Arrays**, **Infinium**, **BeadXpress**, **VeraCode**, **IntelliHyb**, **iSelect**, **CSPro**, **iScan**, and **GenomeStudio** are registered trademarks or trademarks of Illumina. All other brands and names contained herein are the property of their respective owners.

Introduction

This protocol explains how to convert total RNA into a library of template molecules suitable for high throughput DNA sequencing on the Illumina Cluster Station and Genome Analyzer.

The first step in the workflow involves purifying the poly-A containing mRNA molecules using poly-T oligo-attached magnetic beads. Following purification, the mRNA is fragmented into small pieces using divalent cations under elevated temperature. Then the cleaved RNA fragments are copied into first strand cDNA using reverse transcriptase and random primers. This is followed by second strand cDNA synthesis using DNA Polymerase I and RNaseH. These cDNA fragments then go through an end repair process, the addition of a single 'A' base, and then ligation of the adapters. These products are then purified and enriched with PCR to create the final cDNA library.

Workflow

You will need two days to complete this protocol.

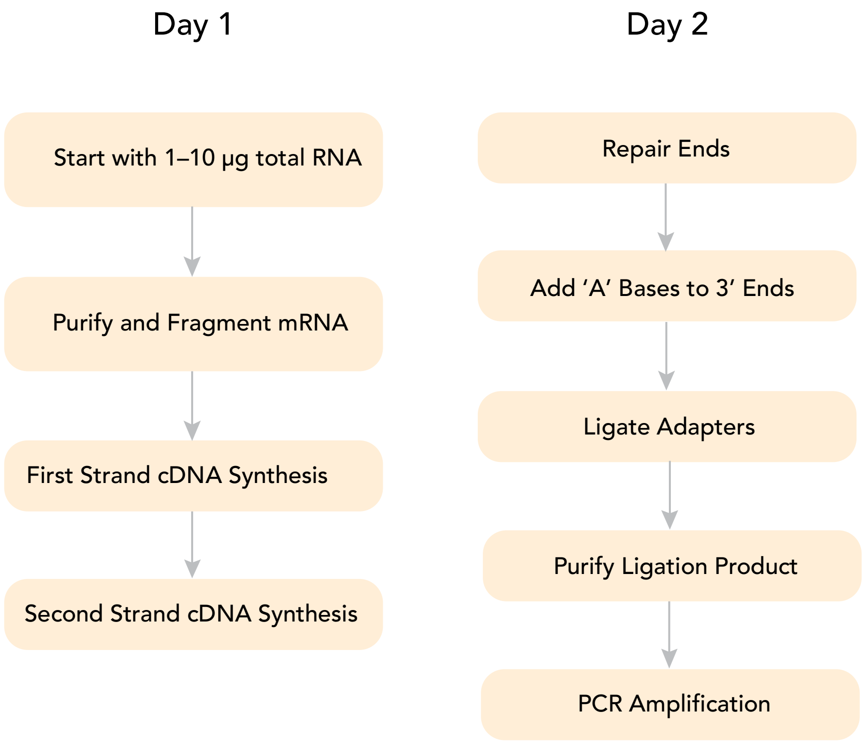

Figure 1 Sample Preparation Workflow

NOTE: The sample may be stored in ethanol precipitation, or after each QIAGEN purification step, if necessary.

Sample Preparation Kit Contents

Check to ensure that you have all of the reagents identified in this section before proceeding to sample preparation.

mRNA-Seq Sample Prep Kit (Box)

Store at -15° to -25°C

This box is shipped at -80°C. As soon as you receive your kit, store the following components at -15° to -25°C.

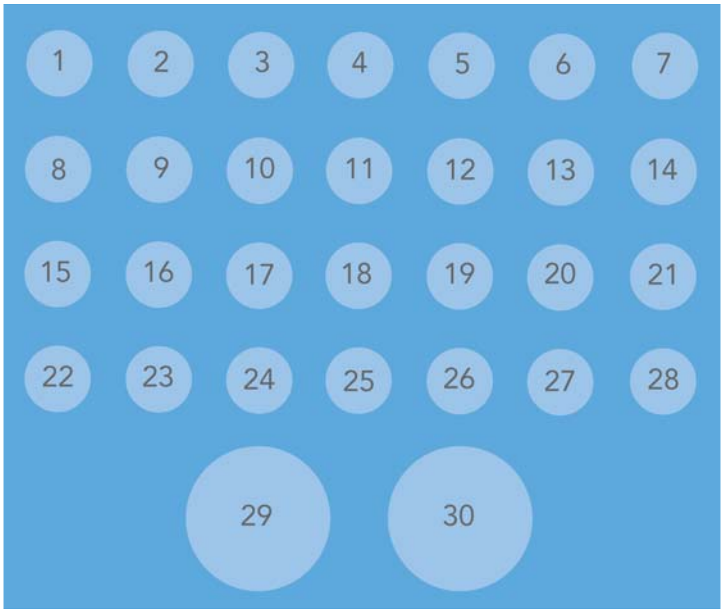

Figure 1 mRNA-Seq Sample Prep Kit, Box 1

1. Ultra Pure Water, part # 1001913
2. 10 mM Tris Buffer, part # 1002115
3. 5X Fragmentation Buffer, part # 1005084
4. Fragmentation Stop Solution, part # 1004826
5. Glycogen, part # 1001664
6. Random Primers, part # 1004784
7. 25 mM dNTPs Mix, part # 11318102
8. RNaseOUT, part # 1000560
9. GEX Second Strand Buffer, part # 1000562
10. RNaseH, part # 1000576
11. DNA Polymerase I, part # 1000577
12. 10X End Repair Buffer, part # 1004819
13. T4 DNA Polymerase, part # 1000514
14. Klenow DNA Polymerase, part # 1000515

15. T4 PNK, part # 1005082
16. 10X A-Tailing Buffer, part # 1002105
17. 1 mM dATP, part # 11318081
18. Klenow Exo -, part # 11318090
19. 2X Rapid T4 DNA Ligase Buffer, part # 1004792
20. PE Adapter Oligo Mix, part # 1001782
21. T4 DNA Ligase, part # 1004790
22. 5X Phusion Buffer, part # 1000585
23. PCR Primer PE 2.0, part # 1001784
24. PCR Primer PE 1.0, part # 1001783
25. Phusion DNA Polymerase (Finnzymes Oy), part # 1000584
26. Empty
27. Empty
28. Empty
29. Bead Binding Buffer, part # 1002118
30. Bead Washing Buffer, part # 1004800

NOTE

Briefly centrifuge the tubes before use, as the contents may have settled on the sides.

mRNA-Seq Sample Prep Kit (Bag)

Store at 2° to 8°C

This bag is shipped at 4°C. As soon as you receive your kit, store the contents at 2° to 8°C.

- Sera-mag Magnetic Oligo(dT) Beads, part # 1004815

User-Supplied Consumables and Equipment

Check to ensure that you have all of the necessary user-supplied consumables and equipment before proceeding to sample preparation.

Consumables

The user-supplied consumables are also listed at the beginning of each section.

- 3 M NaOAC, pH 5.2
- 70% EtOH
- 100% EtOH
- SuperScript II (Invitrogen, part # 18064-014)
    - SuperScript II
    - 100 mM DTT
    - 5X First-Strand Buffer
- Certified Low-Range Ultra Agarose (BIO-RAD, part # 161-3106)
- 50x TAE Buffer
- Distilled Water
- 6X DNA Loading Dye
- 100 bp DNA Ladder (Invitrogen, part # 10488-058)
- QIAquick Gel Extraction Kit (QIAGEN, part # 28704)
- QIAquick PCR Purification Kit (QIAGEN, part # 28104)
- MinElute PCR Purification Kit (QIAGEN, part # 28004)
- GeneCatcher Disposable Gel Excision Kit (Gel Company, part # PKB6.5)

Equipment Checklist

Check to ensure that you have all of the necessary user-supplied equipment before proceeding to sample preparation.

- 4°C microcentrifuge
- Room temperature tube rotator
- Thermal cycler
- Magnetic stand
- Heat block

Purify mRNA from Total RNA

This protocol purifies the poly-A containing mRNA molecules using poly-T oligo-attached magnetic beads.

Illumina recommends that you check total RNA integrity following isolation using an Agilent Technologies 2100 Bioanalyzer with an RNA Integrity Number (RIN) value greater than 8. Alternatively, a formaldehyde 1% agarose gel can be run and the integrity of RNA judged upon staining with ethidium bromide. High quality RNA will show a 28S rRNA band at 4.5 kb that should be twice the intensity of the 18S rRNA band at 1.9 kb. Both kb determinations are relative to a RNA 6000 ladder. The mRNA will appear as a smear from 0.5–12 kb.

Wear gloves and use sterile techniques when working with RNA. All plastic ware and reagents should be RNase-free.

Consumables

Illumina-Supplied

- Sera-mag Magnetic Oligo(dT) Beads
- Bead Binding Buffer
- Bead Washing Buffer
- Ultra Pure Water
- 10 mM Tris Buffer

Best Practice

Using the Magnetic Stand

Follow these guidelines throughout the sample preparation protocol to prevent the beads from drying out.

NOTE

Do not allow the beads to dry during the entire process. During all wash steps, add buffers to the tube containing the beads while the tube is on the magnetic stand.

1. Place the tube containing the beads on the magnetic stand for 1–2 minutes to separate the beads and the buffer.
2. Exchange the buffer using a pipette while the tube is on the magnetic stand.

CAUTION

It is critical that the beads are thoroughly resuspended in the solution.

3. Resuspend the beads thoroughly by vortexing.
4. Repeat steps 1 through 3 as required.

Procedure

It is important to follow this procedure exactly to ensure reproducibility.

NOTE

You may also start this protocol with 100 ng of mRNA. If so, proceed to the next section, *Fragment the mRNA* on page 9.

NOTE

Illumina recommends you dilute 1–10 µg of total RNA.

1. Dilute the total RNA with nuclease-free water to 50 µl in a 1.5 ml RNase-free non-sticky tube.
2. Heat the sample at 65°C for 5 minutes to disrupt the secondary structures and then place the tube on ice.
3. Aliquot 15 µl of Sera-mag oligo(dT) beads into a 1.5 ml RNase-free non-sticky tube.
4. Wash the beads two times with 100 µl of Bead Binding Buffer and remove the supernatant.
5. Resuspend the beads in 50 µl of Bead Binding Buffer and add the 50 µl of total RNA sample from step 2.
6. Aliquot 50 µl of Binding Buffer to a fresh 1.5 ml RNase-free non-sticky Eppendorf tube.
7. Rotate the tube from step 5 at room temperature for 5 minutes and remove the supernatant.
8. Wash the beads twice with 200 µl of Washing Buffer and remove the supernatant.
9. Add 50 µl of 10 mM Tris-HCl to the beads and then heat at 80°C for 2 minutes to elute the mRNA from the beads.
10. Immediately put the tube on the magnet stand, transfer the supernatant (mRNA) to the tube from step 6, and add 200 µl of Washing Buffer to the beads.
11. Heat the samples at 65°C for 5 minutes to disrupt the secondary structures and then place the samples on ice.
12. Wash the beads from step 9 twice with 200 µl of Washing Buffer and remove the supernatant.
13. Add 100 µl of the mRNA sample from step 11 rotate it at room temperature for 5 minutes, and remove the supernatant.
14. Wash the beads twice with 200 µl of Washing Buffer and remove the supernatant.
15. Add 17 µl of 10 mM Tris-HCl to the beads and heat at 80°C for 2 minutes to elute the mRNA from the beads.
16. Immediately put the tube on the magnet stand and then transfer the supernatant (mRNA) to a fresh 200 µl thin-wall PCR tube.

    The resulting amount of mRNA should be approximately 16 µl.

Fragment the mRNA

This protocol fragments the mRNA into small pieces using divalent cations under elevated temperature.

Consumables

Illumina-Supplied

- 5X Fragmentation Buffer
- Fragmentation Stop Solution
- Glycogen
- Ultra Pure Water

User-Supplied

- 3 M NaOAC, pH 5.2
- 70% EtOH
- 100% EtOH

Procedure

1. Prepare the following reaction mix in a 200 µl thin wall PCR tube:
   - 5X Fragmentation Buffer (4 µl)
   - mRNA (16 µl)

   The total volume should be 20 µl.
2. Incubate the tube in a PCR thermal cycler at 94°C for exactly 5 minutes.

   The thermal cycler should be at 94°C before putting the tube in to avoid ramping time.
3. Add 2 µl of Stop Buffer.
4. Place the tube on ice.
5. Transfer the solution to a 1.5 ml microcentrifuge tube.
6. Add the following to the tube and incubate at -80°C for 30 minutes:
   - 3 M NaOAC, pH 5.2 (2 µl)
   - Glycogen (2 µl)
   - 100% EtOH (60 µl)
7. Centrifuge the tube at 14,000 rpm (20,200 relative centrifugal force) for 25 minutes at 4°C in a microcentrifuge.
8. Carefully pipette EtOH without dislodging the RNA pellet.
9. Wash the pellet with 300 µl of 70% EtOH.
10. Centrifuge the pellet and carefully pipette out the 70% EtOH.
11. Air dry the pellet for 10 minutes.
12. Resuspend the RNA in 11.1 µl of RNase-free water.

Synthesize the First Strand cDNA

This protocol copies the cleaved RNA fragments into first strand cDNA using reverse transcriptase and random primers.

Consumables

Illumina-Supplied

- 25 mM dNTP Mix
- Random Primers
- RNaseOUT

User-Supplied

- 100 mM DTT
- SuperScript II (part of SuperScript II)
- 5X First Strand Buffer (part of SuperScript II)

Procedure

1. Assemble the following reaction in a 200 µl thin wall PCR tube:
   - Random Primers (1 µl)
   - mRNA (11.1 µl)

   The total volume should be 12.1 µl.
2. Incubate the sample in a PCR thermal cycler at 65°C for 5 minutes, and then place the tube on ice.
3. Mix the following reagents in the order listed in a separate tube.

   Multiply each volume by the number of samples being prepared. Prepare 10% extra reagent mix if you are preparing multiple samples.
   - 5X First Strand Buffer (4 µl)
   - 100 mM DTT (2 µl)
   - 25 mM dNTP Mix (0.4 µl)
   - RNaseOUT (0.5 µl)

   The total volume should be 6.9 µl.
4. Add 6.9 µl of mixture to the PCR tube and mix well.
5. Heat the sample at 25°C in a thermal cycler for 2 minutes.
6. Add 1 µl SuperScript II to the sample and incubate the sample in a thermal cycler with following program:
   a. 25°C for 10 minutes
   b. 42°C for 50 minutes
   c. 70°C for 15 minutes
   d. Hold at 4°C
7. Place the tube on ice.

Synthesize the Second Strand cDNA

This protocol removes the strand of mRNA and synthesizes a replacement strand generating double-stranded cDNA.

Consumables

Illumina-Supplied

- Ultra Pure Water
- GEX Second Strand Buffer
- 25 mM dNTP Mix
- RNaseH
- DNA Pol I

User-Supplied

- QIAquick PCR Purification Kit (QIAGEN, part # 28104)

Procedure

1. Add 62.8 µl of water to the first strand cDNA synthesis mix.
2. Add the following reagents to the mix:
   - GEX Second Strand Buffer (10 µl)
   - 25 mM dNTP Mix (1.2 µl)
3. Mix well and incubate on ice for 5 minutes or until well-chilled.
4. Add the following reagents:
   - RNaseH (1 µl)
   - DNA Pol I (5 µl)
5. Mix well and incubate at 16°C in a thermal cycler for 2.5 hours.
6. Follow the instructions in the QIAquick PCR Purification Kit to purify the sample and elute in 50 µl of EB.

Perform End Repair

This protocol converts the overhangs into blunt ends using T4 DNA polymerase and Klenow DNA polymerase. The 3' to 5' exonuclease activity of these enzymes removes 3' overhangs and the polymerase activity fills in the 5' overhangs.

Consumables

Illumina-Supplied

- 10X End Repair Buffer
- 25 mM dNTP Mix
- T4 DNA Polymerase
- Klenow DNA Polymerase
- T4 PNK
- Ultra Pure Water

User-Supplied

- QIAquick PCR Purification Kit (QIAGEN, part # 28104)

Procedure

1. Prepare the following reaction mix in a 1.5 ml Eppendorf tube:
   - Eluted DNA (50 µl)
   - Water (27.4 µl)
   - 10X End Repair Buffer (10 µl)
   - 25 mM dNTP Mix (1.6 µl)
   - T4 DNA Polymerase (5 µl)
   - Klenow DNA Polymerase (1 µl)
   - T4 PNK (5 µl)

   The total volume should be 100 µl.
2. Incubate the sample in a heat block at 20°C for 30 minutes.
3. Follow the instructions in the QIAquick PCR Purification Kit to purify the sample and elute in 32 µl of EB.

Add 'A' Bases to the 3' End of the DNA Fragments

This protocol adds an 'A' base to the 3' end of the blunt phosphorylated DNA fragments, using the polymerase activity of Klenow fragment (3' to 5' exo minus). This prepares the DNA fragments for ligation to the adapters, which have a single 'T' base overhang at their 3' end.

Consumables

Illumina-Supplied

- A-Tailing Buffer
- 1 mM dATP
- Klenow Exo -

User-Supplied

- MinElute PCR Purification Kit (QIAGEN, part # 28004)

NOTE This protocol requires a MinElute column rather than a normal QIAquick column.

Procedure

1. Prepare the following reaction mix in a 1.5 ml Eppendorf tube:
   - Eluted DNA (32 µl)
   - A-Tailing Buffer (5 µl)
   - 1 mM dATP (10 µl)
   - Klenow Exo -

   The total volume should be 50 µl.
2. Incubate the sample in a heat block at 37°C for 30 minutes.
3. Follow the instructions in the MinElute PCR Purification Kit to purify the sample and elute in 23 µl of EB.

Ligate Adapters to DNA Fragments

This protocol ligates adapters to the ends of the DNA fragments, preparing them to be hybridized to a flow cell.

Consumables

Illumina-Supplied

- 2X Rapid T4 DNA Ligase Buffer
- PE Adapter Oligo Mix
- T4 DNA Ligase

User-Supplied

- MinElute PCR Purification Kit (QIAGEN, part # 28004)

Procedure

1. Prepare the following reaction mix in a 1.5 ml Eppendorf tube:
   - Eluted DNA (23 µl)
   - 2X Rapid T4 DNA Ligase Buffer (25 µl)
   - PE Adapter Oligo Mix (1 µl)
   - T4 DNA Ligase (1 µl)

   The total volume should be 50 µl.
2. Incubate the sample at room temperature for 15 minutes.
3. Follow the instructions in the MinElute PCR Purification Kit to purify the sample and elute in 10 µl of EB.

Purify cDNA Templates

This protocol purifies the products of the ligation reaction on a gel to select a size range of templates for downstream enrichment.

Consumables

User-Supplied

- Certified Low-Range Ultra Agarose
- 50X TAE Buffer
- Distilled Water
- 100 bp DNA Ladder
- 6X DNA Loading Dye
- GeneCatcher Disposable Gel Excision Kit (Gel Company, part # PKB6.5)
- QIAquick Gel Extraction Kit (QIAGEN, part # 28704)

Procedure

1. Prepare a 50 ml, 2% agarose gel with distilled water and TAE. Final concentration of TAE should be 1X at 50 ml.
2. Load the samples as follows:
   - 2 µl 100 bp DNA Ladder in the first well
   - 10 µl DNA elute from the ligation step mixed with 2 µl of 6X DNA Loading Dye in the second well
   - 2 µl 100 bp DNA Ladder in the third well

   Using ladders on both sides of a sample help locate the gel area to be excised as the band will not be visible.

   NOTE For handling multiple samples, leave one empty lane between samples and ladders to prevent cross-contamination. Do not run more than two samples on the same gel to avoid contamination.

3. Run the gel at 120 V for 60 minutes.
4. Excise a region of gel with a clean gel excision tip and remove the gel slice by centrifuging it into a microcentrifuge tube. The gel slice should contain the material in the 200 bp (±25 bp) range. See Figure 1.

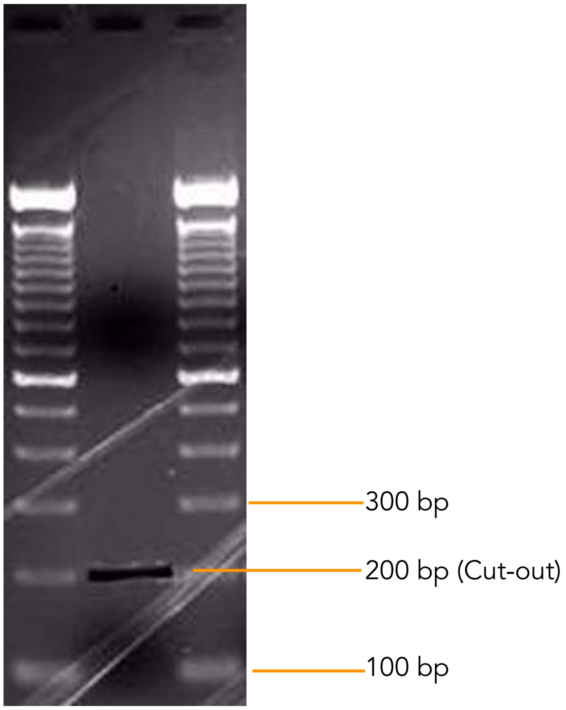

Figure 1 *2% Agarose Gel*

5. Follow instructions in the QIAquick Gel Extraction Kit to purify the sample and elute in 30 µl of EB. (Be sure to add isopropanol per the manufacturer's instructions.)

It is normal to not see any visible DNA on the gel.

Enrich the Purified cDNA Templates by PCR

This protocol uses PCR to amplify the cDNA in the library. The PCR is performed with two primers that anneal to the ends of the adapters.

Consumables

Illumina-Supplied

- 5X Phusion Buffer
- Phusion DNA Polymerase
- PCR Primer PE 1.0
- PCR Primer PE 2.0
- 25 mM dNTP Mix
- Ultra Pure Water

User-Supplied

- QIAquick PCR Purification Kit (QIAGEN, part # 28104)

Procedure

1. Prepare the following PCR reaction mix in a 200 µl thin wall PCR tube (Make 10% extra reagent for multiple samples):
   - 5X Phusion Buffer (10 µl)
   - PCR Primer PE 1.0 (1 µl)
   - PCR Primer PE 2.0 (1 µl)
   - 25 mM dNTP Mix (0.5 µl)
   - Phusion DNA Polymerase (0.5 µl)
   - Water (7 µl)

   The total volume should be 20 µl.
2. Add 30 µl of purified ligation mix (from step 5 of the previous section) to the 200 µl PCR tube.
3. Amplify using the following PCR protocol:
   - **a.** 30 seconds at 98°C
   - **b.** 15 cycles of:
     10 seconds at 98°C
     30 seconds at 65°C
     30 seconds at 72°C
   - **c.** 5 minutes at 72°C
   - **d.** Hold at 4°C
4. Follow the instructions in the QIAquick PCR Purification Kit to purify the sample and elute in 30 µl of EB.

Validate the Library

Illumina recommends performing the following quality control analysis on your sample library to quantify the DNA concentration.

1. Load 1 µl of the resuspended construct on an Agilent Technologies 2100 Bioanalyzer using the Agilent DNA 1000 chip kit (Agilent, part # 5067-1504).
2. Check the size, purity, and concentration of the sample. The final product should be a distinct band at approximately 200 bp.

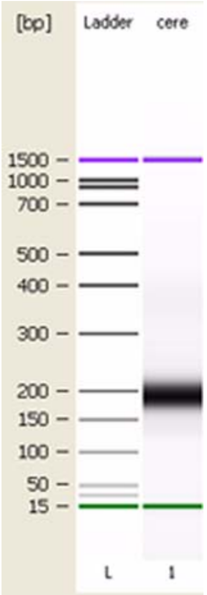

Figure 1 200 bp PCR Product

[Optional] You can confirm the final product by cloning 1 µl of the product into Invitrogen Zero Blunt TOPO vector, and sequence using conventional technology.

Illumina, Inc.
9885 Towne Centre Drive
San Diego, CA 92121-1975
+1.800.809.ILMN (4566)
+1.858.202.4566 (outside North America)
techsupport@illumina.com
www.illumina.com
